# Genistein enhances anti-PD-1 efficacy in melanoma by suppressing regulatory T cell differentiation and activity

**DOI:** 10.1038/s41598-025-20941-7

**Published:** 2025-10-22

**Authors:** Fei Mo, Min Hong, Xiao Chen, Qiye Wang, Chao Dong

**Affiliations:** Department of Medical Oncology, The First Affiliated Hospital of Kunming Medical University, NO.295 Xichang Road, Kunming, 650032 Yunnan China

**Keywords:** Genistein, Anti-PD-1, Regulatory t (Treg) cell, PI3K/AKT, Melanoma, Cell biology, Molecular biology

## Abstract

The accumulation of regulatory T cells (Tregs) in the tumor microenvironment is a key factor for immunosuppression and immune escape of cancer cells. Genistein is a natural isoflavonoid in soybeans, possessing a wide range of pharmacological bioactivities. However, the immunomodulatory potential of genistein remains largely unexplored. This study investigated the synergistic efficacy of genistein with anti-PD-1 therapy in a mouse model of melanoma. The regulatory role of genistein in Treg differentiation and functional stability was also examined in this study. The antitumor effects of genistein, alone or in combination with anti-PD-1 therapy, were evaluated in the mouse B16-F10 melanoma model. The profiles of intratumoral Tregs and CD8^+^ cytotoxic T cells were profiled using flow cytometry. The impact of genistein on the suppressive function and differentiation of Tregs was also studied in vitro. RT-qPCR, Western blotting and flow cytometry were conducted to characterize the functional phenotype of Tregs under genistein treatment. Genistein showed a synergistic effect with anti-PD-1 therapy in B16-F10 melanoma models, achieving enhanced immune infiltration and functional reprogramming of the tumor microenvironment. This combinatorial strategy not only reduced intratumoral Tregs but also elevated the CD8^+^/Treg ratio. At the mechanistic level, genistein effectively suppressed critical Treg surface markers, which are indispensable for Treg-mediated immunosuppression. Furthermore, it disrupted Treg differentiation by attenuating PI3K/AKT signaling phosphorylation. Our data demonstrated that genistein boosts the antitumor effect of anti-PD-1 therapy by impairing the immunosuppressive function and differentiation of Tregs, indicating that genistein has the potential to enhance the treatment outcome of the immune checkpoint inhibitor.

**Supplementary Information:**

The online version contains supplementary material available at 10.1038/s41598-025-20941-7.

## Introduction

Tumor microenvironment involves the complex interactions of cancer cells, stromal cells and immune cells. The infiltration of immunosuppressive cells and related cytokines suppresses the tumoricidal activity of CD8^+^ cytotoxic T cells^1^. Regulatory T cells (Tregs), a subset of CD4^+^ T lymphocytes defined by the expression of the transcription factor Foxp3, exert immunosuppressive effects to suppress excessive immune activation and preserve immune homeostasis^2^. Tregs actively infiltrate the tumor microenvironment and adapt in the nutrient-deprived environment by metabolic reprogramming^3,4^. Besides, Tregs express high levels of immune checkpoint receptors, such as CTLA4, PD-1, Tim-3, TIGIT and LAG-3, through which Tregs impair the activity of antigen-presenting cells (APCs) and effector T cells^5–7^.

The discovery of immune checkpoints has opened new avenues to counteract immunosuppression and reinvigorate anti-tumor immunity within the tumor microenvironment^8^. The application of antibodies or inhibitors against PD-1 or PD-L1 has demonstrated a potent effect in unleashing the tumoricidal potential of immune cells^9^. However, in solid tumors, the accumulation of immunosuppressive Tregs compromises antitumor immune responses, thereby diminishing the clinical benefits of immune checkpoint inhibitors^10,11^. Selective depletion of Tregs has been proposed as a promising strategy to enhance antitumor immune responses induced by immune checkpoint inhibitors when combined with other therapies^12^.

Genistein is a natural isoflavone isolated from soybean and possesses diverse bioactivities. Emerging evidence demonstrates that genistein exerts anti-inflammatory effects and ameliorates disease progression in diverse pathological contexts, such as encephalomyelitis (EAE), non-alcoholic steatohepatitis (NASH), and endometriosis^13,14^. Furthermore, the chemopreventive effect of genistein has been widely reported in multiple cancer types^15,16^, including melanoma^17^, breast cancer^18^, prostate cancer^19^, ovarian cancer^20^ and colorectal cancer^21^. However, current evidence on genistein’s role in modulating the tumor immune microenvironment (TIME) remains limited.

This study aimed to evaluate the immunomodulatory potential of genistein in the microenvironment of melanoma. We demonstrated that genistein treatment alone exerts therapeutic effects in C57BL/6 melanoma-bearing mice. Notably, the combination of genistein and anti-PD-1 therapy enhanced synergistic antitumor responses by reduction in Treg-mediated immunosuppression and increased CD8^+^ T cell infiltration. Mechanistic investigations revealed that genistein impairs the suppressive function and differentiation of Tregs by attenuating the PI3K/AKT signaling pathway.

## Methods

### Cell line culture

Murine melanoma cells (B16-F10) were purchased from RiCells Biotech (ORC0027, Shanghai, China). The cells were cultured in RPMI-1640 medium (Procell, Wuhan, China) supplemented with 10% fetal bovine serum (FBS, Sigma, Shanghai, China) and 1% penicillin/streptomycin (Procell, Wuhan, China) at 37 °C with 5% CO_2_.

### Mice

Eight-week-old female C57BL/6 mice were purchased from Weitong Lihua Experimental Animal Technology Co., Ltd. (Beijing, China) and housed under specific-pathogen-free (SPF) conditions at Kunming Medical University, with free access to food and water (temperature:21–25 °C; humidity: 30–65%; light/dark cycle: 12 h/12 h). The mice were euthanized by CO_2_ inhalation when the tumor volume reached 1500 mm^2^ or when they showed poor condition and were expected to die soon. All animal experiments were approved by the Animal Ethics Committee of Kunming Medical University (kmmu20230475). All methods were performed in accordance with the relevant guidelines and regulations. The study is reported in accordance with ARRIVE guidelines.

### Primary cell isolation and culture

Spleens were collected from euthanized mice and homogenized in phosphate-buffered saline (PBS) containing 5% FBS. The cell suspension was filtered through a 40 μm cell strainer, and splenocytes were isolated using the MagCellect Mouse CD4^+^ CD25^+^ Regulatory T Cell Isolation Kit (MAGM208, R&D systems, CA, USA) to obtain primary CD4^+^ T lymphocytes and CD4^+^CD25^+^ Tregs. First, CD4^+^ T cells were purified from splenocytes using the MagCellect Mouse CD4^+^ T Cell Biotinylated Antibody Cocktail. Then CD4^+^CD25^+^ cells were isolated from the pre-enriched cells using Anti-Mouse CD25 Biotinylated Antibody. Isolated primary T cells were cultured in RPMI-1640 medium supplemented with 10% FBS, 50 µg/mL gentamicin, 50 µM 2-mercaptoethanol (Procell, Wuhan, China) and 10 ng/ml recombinant murine IL-2 (P5907, Beyotime, Beijing, China). To assess the dose-dependent effects of genistein (HY-14596, MedChemExpress, Shanghai, China), Tregs were treated with graded concentrations of genistein (0, 1, 5, 10, 20 µM; HY-N0595, MedChemExpress) for 24 h. Cells were then harvested for quantitative PCR analysis of Treg-related transcripts (Foxp3, CTLA-4, LAG-3), Western blotting detection of Foxp3 expression. Supernatants were collected for quantification of IL-10 by ELISA.

### Treg suppression assays

For Treg suppression assays, the isolated CD4^+^CD25^−^ T cells were labeled with 10 µM Carboxyfluorescein succinimidyl ester (CFSE, 21888, Sigma, Shanghai, China) in PBS at 37 °C for 15 min. After staining, the cells were washed twice with PBS before being suspended in RPMI-1640 medium. The labeled cells were then inoculated at 2 × 10^5^ cells per well in U-bottomed 24-well plates coated with αCD3ε (0.6 µg/ml) and soluble αCD28 (1 µg/ml).Tregs were added at graded ratios (1:1 to 1:2), and the co-culture system was maintained at 37 °C, 5%CO_2_ for 72 h, and the proliferation of CD4^+^CD25^−^ T cells was assessed by CFSE dilution using an LSRII flow cytometer (BD Biosciences, CA, USA).

### In vitro Treg differentiation

Isolated CD4^+^CD25^−^ T lymphocytes were subjected to the Treg differentiation assay. The cells were seeded at 2 × 10^5^ cells/well in U-bottom 24-well plate (Corning) pre-coated with αCD3ε antibody (0.6 µg/ml, Clone 145-2C11, eBioscience, CA, USA) and cultured in RPMI-1640 medium containing soluble αCD28 (1 µg/ml, Clone, 37.51 eBioscience), 2 ng/ml TGF-β1 (Meilun Biotech, Beijing, China), 10% FBS, 50 µg/ml gentamicin, 50 µM 2-mercaptoethanol (Procell, Wuhan, China) for 72 h. For chemical intervention, cells were treated with genistein (10 µM) and SC-79 (10 µM, an AKT phosphorylation activator, HY-18749, MedChemExpress) during differentiation.

### In vivo genistein or anti-PD-1 treatments

B16-F10 melanoma cells (5 × 10^6^ cells in 100 µl PBS/mouse) were subcutaneously implanted into the right flank of 8-week-old female C57BL/6 mice under isoflurane anesthesia. When tumor volume reached approximately 200 mm^2^, mice were randomly assigned to treatment groups receiving genistein (160 mg/kg, a dose reported in human clinical trials)^22,23^, anti-PD-1 (InVivoMAb anti-mouse PD-1 (CD279), BE0146, Bio X Cell, 3 mg/kg), or the combination therapy of genistein and anti-PD-1. Mice were injected with PBS as control group. Tumor length (L) and width (W) were measured every other day, and tumor volumes (V) was calculated using the formula: V = L × W^2^ × 0.52. After euthanasia, tumors were collected for subsequent TUNEL staining, ELISA quantification, and multiparametric flow cytometry analysis.

### Terminal deoxynucleotidyl transferase (TdT) dUTP nick-end labeling (TUNEL)

TUNEL staining was performed on 5 μm-thick formaldehyde-fixed, paraffin-embedded (FFPE) tissue sections. After deparaffinization and rehydration, apoptosis was detected using the In Situ Cell Death Detection Kit, AP (11684809910, Roche) according to the manufacturer’s instructions. The images were captured using a Leica DM6000 microscope (Leica, Wetzlar, Germany).

### ELISA

Tumor tissues were homogenized in ice-cold PBS containing a protease inhibitor cocktail (1:100 dilution, HZ-5029, Sangon Biotechnology, Shanghai, China), followed by centrifugation at 12,000 × g for 15 min at 4 °C to collect the supernatant. For cell samples, culture supernatants were harvested and centrifuged at 2000 × g for 10 min at 4 °C to remove cell debris. The concentrations of IFN-γ, TNF-α, and IL-10 in the supernatants were measured using corresponding ELISA kits (Biovision, CA, USA), following the manufacturer’s protocols. Absorbance value at 450 nm was recorded using a microplate photometer (HiPo MPP-96, Biosan, Riga, Latvia) and concentrations were calculated based on standard curves.

### Western blotting

After removing culture supernatants, Tregs were lysed in RIPA buffer (Beyotime Biotechnology, P0045) containing a protease inhibitor cocktail (HZ-5029, Sangon Biotechnology, Shanghai, China) and boiled for 10 min. Protein concentrations were quantified using a protein colorimetric assay kit (ZY610021RE, Zeye Biotech, Shanghai, China) according to the manufacturer’s protocol. Proteins (20 µg/lane) were separated by 12.5% SDS‒PAGE and transferred onto PVDF membranes (Millipore). Then, the PVDF membranes were incubated with primary antibodies (1:1000 dilution, 4 °C overnight), followed by horseradish peroxidase-conjugated secondary antibodies at room temperature for 1 h. Bands were visualized using BeyoECL Plus chemiluminescent reagent (P0018FS, Beyotime, Beijing, China) and detected with a ChemiDoc MP Imaging System (Bio-Rad). Protein band intensities were quantified using ImageJ software (Version 1.53t, National Institutes of Health, Bethesda, MD, USA). The following primary antibodies(Abcam, Cambridge, UK) were used: anti-β-actin mAb (ab8227), anti-Foxp3 mAb (ab215206), anti-AKT mAb (ab18785), anti-p-AKT mAb (ab38449), anti-PI3K mAb (ab191606) and anti-p-PI3K mAb (ab182651).

### RNA isolation and real-time quantitative PCR

Total RNA was extracted from Tregs using a mammalian cell mRNA isolation kit (ZY80817, Zeye Biotech, Shanghai, China). RNA was reverse-transcribed into cDNA using the PrimeScript™ RT Reagent Kit (RR037B, Takara, Dalian, China). Quantitative PCR was performed on a 7500 Real Time PCR System (Applied Biosystems, CA, USA) using the SYBR premix EX TAQ II kit (RR820A, Takara, Dalian, China). All PCR primers were provided by Sangon Biotechnology (Shanghai, China). Relative gene expression levels were calculated using the 2 ^−ΔΔCt^ method, with β-actin mRNA as the reference gene.

### Flow cytometry

Tumor tissues were minced into 1–2 mm^2^ pieces and transferred to RPMI 1640 medium containing a digestive enzyme mixture (collagenase IV (HY-E70005D, MedChemExpress, USA) 1 mg/ml, DNase I (D755017-100 mg, aladdin, Shanghai, China) 0.1 mg/ml, and hyaluronidase (H754463, aladdin, Shanghai, China) 0.1 mg/ml) for digestion at 37 °C for 60 min, with gentle stirring every 15 min. The tissue suspension was filtered through a 70 μm cell strainer, centrifuged at 400×g for 5 min, and washed twice with PBS containing 2% FBS. Viable cells were isolated by density gradient centrifugation (Ficoll-Paque) and washed again with PBS containing 2% FBS prior to staining. For cell surface staining, cells were incubated with Fc-Block (αCD16/32, 1:500) at 4 °C for 10 min, followed by washing with PBS containing 2% FBS. Cells were then stained with fluorophore-conjugated antibodies (PE-αCD25, 1:600; PB-αCD4, 1:400; FITC-αCD3, 1:400; APC-αCD8, 1:400) at 4 °C for 15 min. For intracellular staining, cells were fixed and permeabilized using the Foxp3 Transcription Factor Staining Buffer Set (421403, BioLegend, San Diego, CA, USA) and subsequently stained with APC-αFoxp3 (1:100). After three washes with permeabilization buffer, cells were resuspended in PBS and analyzed on an LSRII flow cytometer (BD Biosciences, San Jose, CA, USA).

### Statistical analysis

Statistical analyses were performed using SPSS software (Version 19.0, IBM, Armonk, NY, USA). Results are presented as mean ± standard deviation (SD). Data were analyzed using unpaired t-test for comparisons between two groups. For multiple group comparisons, one-way analysis of variance (ANOVA) followed by Tukey’s post hoc test was performed. Statistical significance was defined as *p* < 0.05, denoted as: ns (not significant), *p < 0.05, **p < 0.01, ***p < 0.001.

## Results

### Genistein and anti-PD-1 combination therapy effectively inhibited tumor growth in mice

**Fig. 1:**
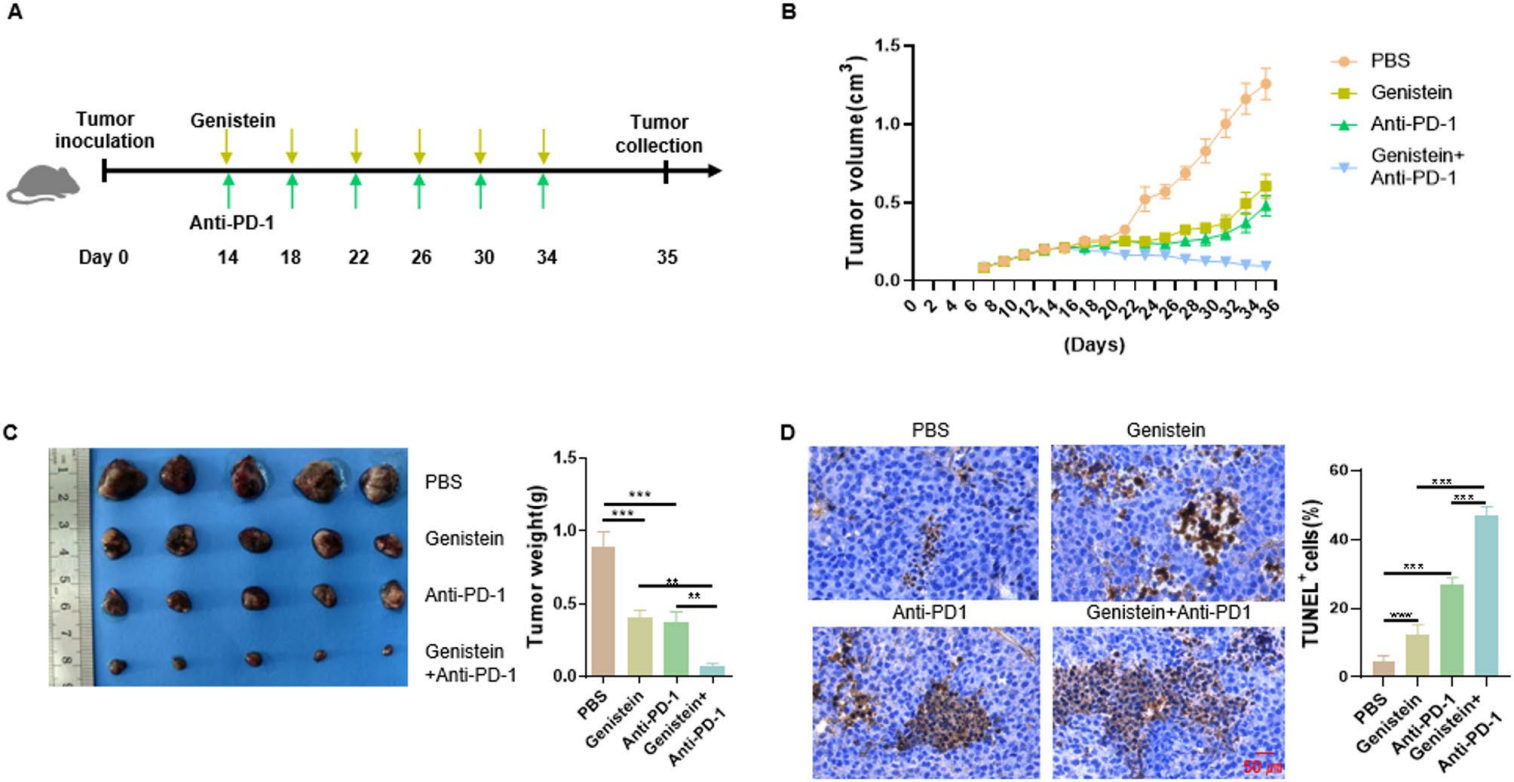
Genistein and anti-PD-1 combination therapy effectively inhibited tumor growth in mice. Following the implantation of B16-F10 tumors, mice were treated with genistein (160 mg/kg) and anti-PD-1 antibodies (3 mg/kg). (**A**) Timeline for tumor inoculation and drug administration. (**B**) Tumor volumes at indicated time points (*n* = 5). (**C**) Tumor imaging with corresponding weight quantification for each treatment group. (**D**) Quantification of apoptotic cells using TUNEL staining in tumor specimens after murine euthanasia and tissue processing. Data are showed as mean ± SD. (*n* = 5); *p < 0.05; **p < 0.01; ***p < 0.001.

The timeline for tumor inoculation and drug administration is shown in Fig. 1A. B16-F10 cells were subcutaneously implanted into the left flank of C57BL/6 mice. Mice received intraperitoneal injections of genistein on days 14, 18, 22, 26, 30, and 34 post-tumor implantation. Additionally, anti-PD-1 monoclonal antibodies were administered intravenously on the same days. As demonstrated in Fig. 1B,C, the combination of genistein and anti-PD-1 significantly retarded tumor growth. The treatment of genistein or anti-PD-1 alone elicited only partial antitumor effect in the murine tumor model. TUNEL assays, which detect apoptotic cells by labeling DNA breaks, revealed that combination of genistein and anti-PD-1 therapy markedly enhanced apoptotic cell death compared to monotherapy (Fig. 1D). These findings demonstrated the synergistic antitumor efficacy of genistein combined with anti-PD-1 therapy.

### Genistein reversed the immunosuppressive microenvironment in melanoma

**Fig. 2:**
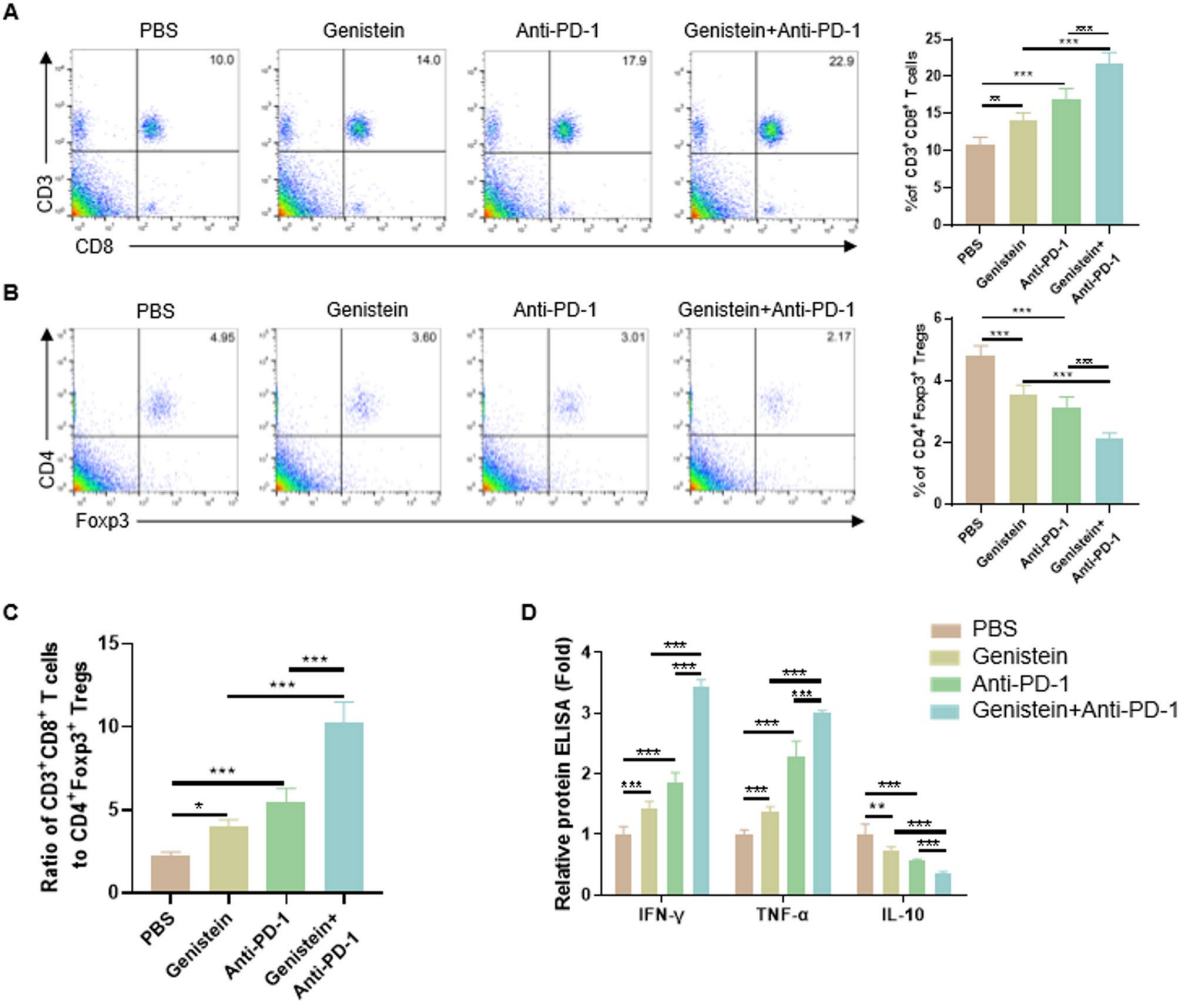
Genistein reversed the immunosuppressive microenvironment in melanoma. (**A**) Quantification of tumor-infiltrating CD3⁺CD8⁺ cytotoxic T lymphocytes (Gating Strategy Fig. S1). (**B**) Quantification of tumor-infiltrating CD4^+^Foxp3^+^ Tregs. (**C**) Ratio of CD8^+^ cytotoxic T cells to Tregs. (**D**) Concentrations of IFN-γ, TNF-α, and IL-10 in tumor tissues measured by ELISA. (*n* = 5). Data are presented as mean ± SD. *p < 0.05; **p < 0.01; ***p < 0.001.

To investigate the immune microenvironment in melanoma, we analyzed CD8^+^ cytotoxic T cells and Tregs in tumor tissues. As shown in Fig. 2A,B, genistein treatment significantly increased CD8^+^ T cell infiltration while reducing Treg populations. The combination of genistein and anti-PD-1 further amplified these effects, resulting in a markedly elevated CD8^+^/Treg ratio compared to either treatment alone (Fig. 2C). The relative levels of key cytokines (IFN-γ, TNF-α, and IL-10) in tumor microenvironments were also quantified. Monotherapy with either genistein or anti-PD-1 therapy moderately increased pro-inflammatory cytokines (IFN-γ and TNF-α) and partially suppressed the expression of the immunosuppressive cytokine IL-10. Notably, the genistein/anti-PD-1 co-treatment group showed amplified cytokine modulation (Fig. 2D). These results indicated that genistein enhanced anti-PD-1 immunotherapy by favorably reshaping the intratumoral immune landscape.

### Genistein dose-dependently impaired Treg function

**Fig. 3:**
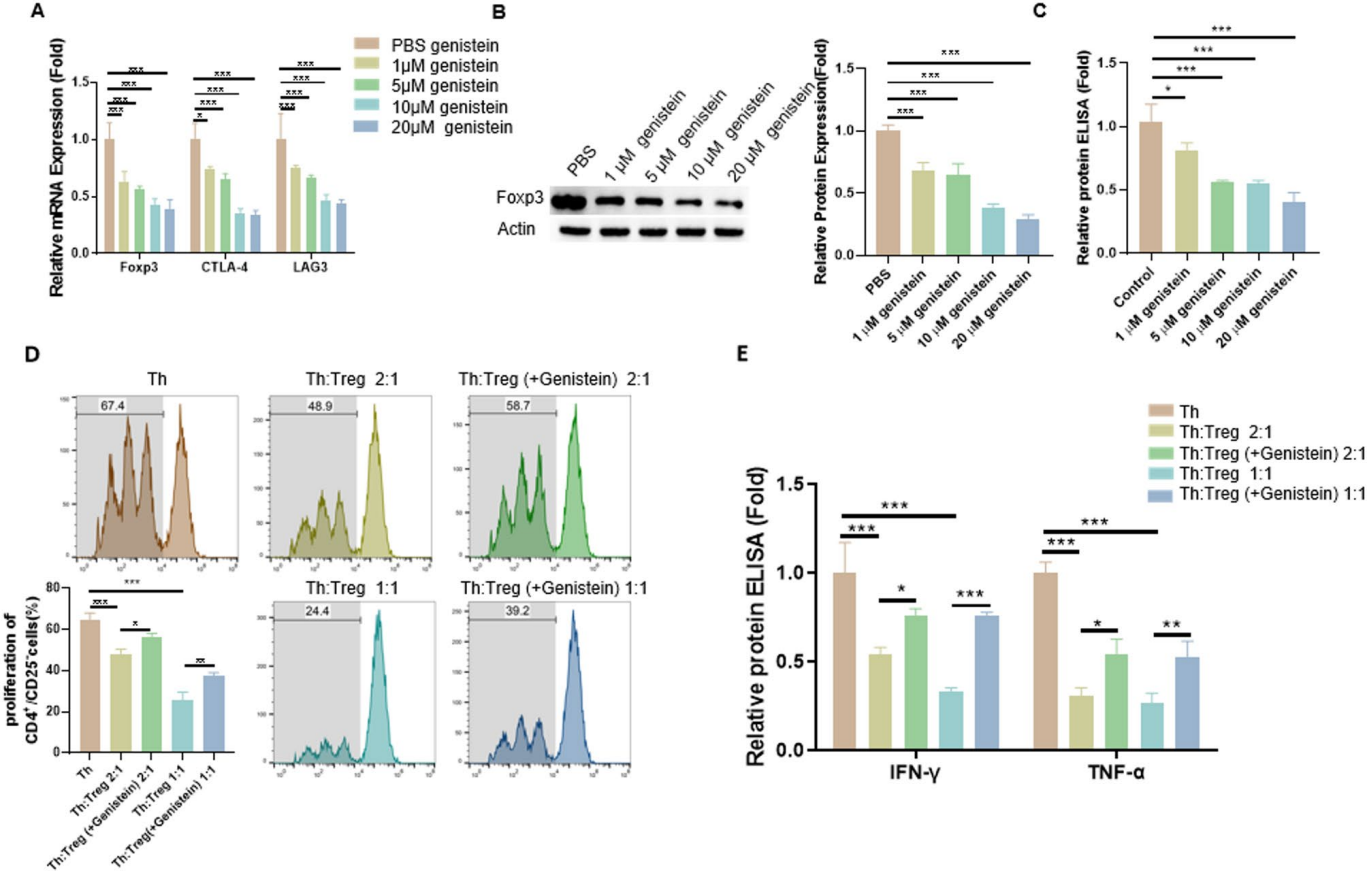
Genistein dose-dependently impaired Treg function. (**A**) RT-qPCR analysis of Treg functional markers (Foxp3, CTLA-4, and LAG-3) after 24-h treatment with genistein at concentrations of 0–20 µM. (**B**) Western blot detection of Foxp3 protein expression following 24-h exposure to genistein (0–20 µM). (**C**) ELISA quantification of IL-10 in Treg culture supernatants after 24-hour genistein treatment (0–20 µM). (**D**) CD4^+^CD25^−^ Th cells (2 × 10^5^/ml) were labeled with CFSE and subsequently cocultured with Tregs preincubated with genistein (10 µM) for 48 h. The Th cell proliferation was determined by flow cytometry. (**E**) ELISA quantification of IFN-γ and TNF-α in supernatants from Treg and Th cell co-cultures. (*n* = 3). Data are showed as mean ± SD; *p < 0.05; **p < 0.01; ***p < 0.001.

To examine the direct effects of genistein on Tregs, CD4^+^ CD25^+^ Tregs were isolated from mouse spleens and treated with increasing concentrations of genistein (1–20 µM). RT-qPCR analysis revealed a dose-dependent reduction in mRNA expression of key Treg functional markers, including Foxp3, CTLA-4, and LAG-3 (Fig. 3A). Western blotting demonstrated a dose-dependent downregulation of Foxp3 protein in genistein-treated Tregs (Fig. 3B). Genistein treatment also reduced IL-10 production by Tregs in a concentration-dependent manner (Fig. 3C). Treg suppressive capacity was assessed by measuring CFSE dilution in T helper (Th) cells. CFSE-labeled CD4^+^CD25^−^ Th cells were co-cultured with Tregs pre-treated with or without 10 µM genistein. As a result, genistein pre-treatment attenuated Treg-mediated suppression of Th cell proliferation (Fig. 3D). Furthermore, genistein pre-treatment impaired Treg suppression of IFN-γ and TNF-α production by Th cells in co-culture (Fig. 3E). These findings collectively demonstrated that genistein significantly impaired the immunosuppressive effect of Tregs.

### Genistein suppressed Treg differentiation by targeting the PI3K-AKT pathway

**Fig. 4:**
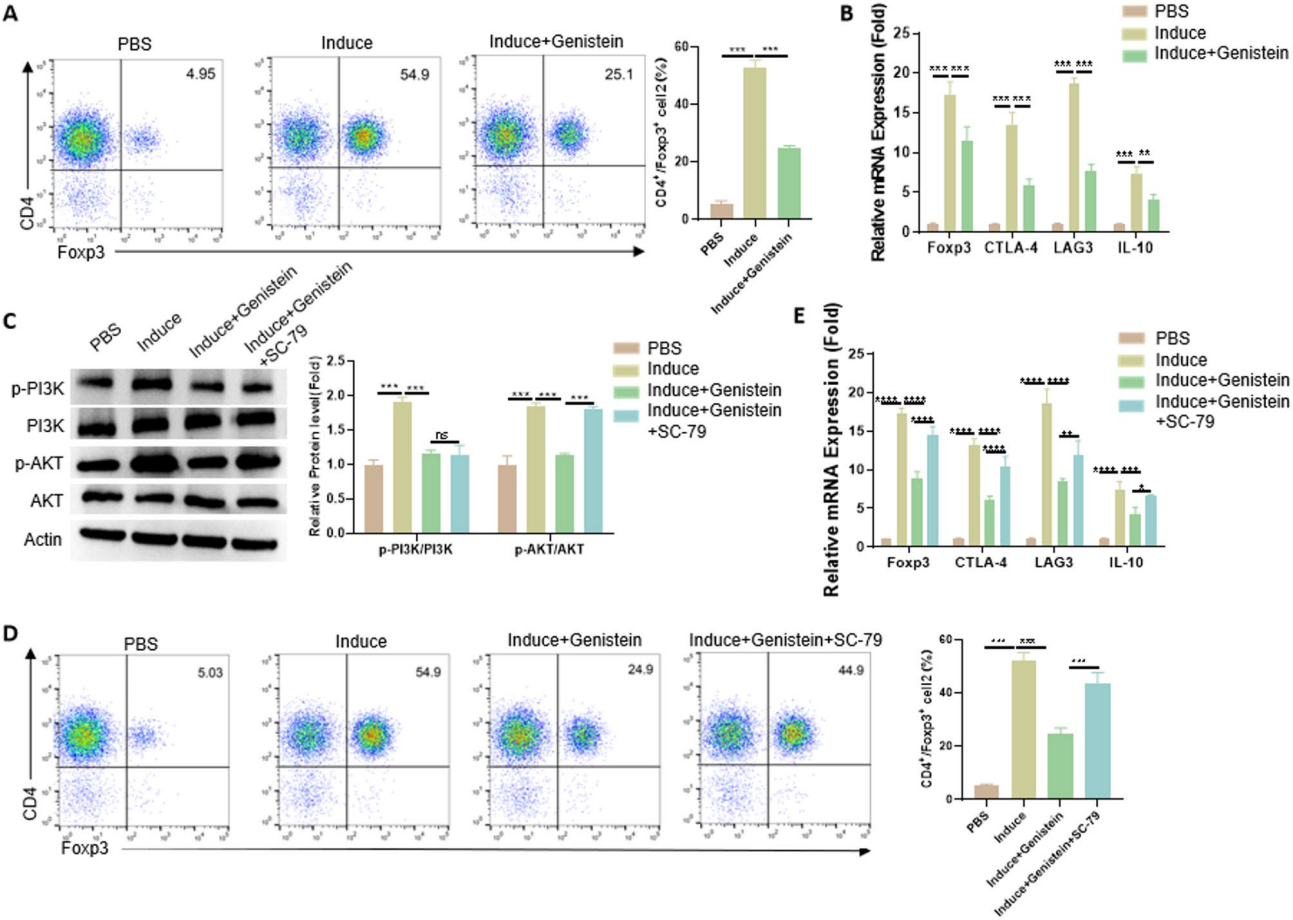
Genistein suppressed Treg differentiation by targeting the PI3K-Akt pathway. (**A**) CD4^+^CD25^−^ naïve T cells were differentiated into Tregs under anti-CD3/CD28 stimulation and 2 ng/ml TGF-β1 for 72 h, with or without 10 µM genistein. Flow cytometry was used to quantify CD4^+^Foxp3^+^ cells. (**B**) qRT-PCR analysis of Treg signature genes (*Foxp3*,* CTLA-4*,* LAG-3*,* IL-10*) in cells treated as in (**A**). (**C**) Western blot detection of phosphorylated PI3K and AKT in the induced T cells, which were incubated with genistein (10 µM) or the combination of genistein (10 µM) and SC-79 (10 µM) under Treg-inducing conditions for 72 h. (**D**) Flow cytometric quantification of CD4^+^Foxp3^+^ cells under the conditions in (**C**). (**E**) mRNA expression of Foxp3, CTLA-4, LAG-3 and IL-10 in induced T cells under the conditions in (**C**). Data are presented as mean ± SD (*n* = 3); *p < 0.05; **p < 0.01; ***p < 0.001.

To explore whether genistein affects Treg differentiation, the CD4^+^CD25^−^ naïve T cells isolated from spleens were induced to differentiate into Tregs with CD3/CD28 co-stimulation and TGF-β1 induction. Genistein treatment significantly suppressed Treg differentiation in vitro, as evidenced by a marked reduction in CD4^+^Foxp3^+^ cell frequency (Fig. 4A). Genistein also downregulated mRNA expression of Treg-related markers in the induced T cells, including Foxp3, CTLA-4, LAG-3, and IL-10 (Fig. 4B). Given the role of the PI3K/AKT signaling pathway in Treg induction^24^, we hypothesized that genistein inhibits Treg differentiation by modulating this pathway. Western blotting analysis showed increased p-PI3K and p-AKT expression during Treg differentiation, which was reduced by genistein treatment. However, the AKT phosphorylation activator SC-79 restored p-AKT expression (Fig. 4C). As shown in Fig. 4D, genistein-induced reduction of CD4^+^Foxp3^+^ Treg cells was reversed by SC-79 treatment. Additionally, the mRNA expression of Treg markers (Foxp3, CTLA-4, LAG-3 and IL-10) was largely restored in the induced T cells by SC-79 treatment (Fig. 4E). These findings demonstrated that genistein inhibited Treg differentiation by suppressing the PI3K/AKT signaling pathways.

## Discussion

Genistein, a natural polyphenolic isoflavone, has been widely evaluated for its protective benefits in the treatment of various cancer types^25^. It has been shown to enhance the anticancer effects of certain chemotherapeutic agents. For instance, genistein augments the antiproliferative effects of 5-fluorouracil and cisplatin in cervical and pancreatic cancer cell lines^26,27^. In a rat breast tumor model, long-term genistein supplementation enhanced tamoxifen’s therapeutic response and reduced tamoxifen resistance^28^. However, studies on genistein’s immunomodulatory activity contributing to its anticancer efficacy are limited. This study demonstrated that genistein inhibited Treg immunosuppressive function and differentiation, which may provide new clues to support it as a promising adjuvant for cancer immunotherapy.

The accumulation of immunosuppressive cells, such as myeloid-derived suppressive cells (MDSCs), macrophages, and Tregs, are obstacles for effective anti-tumor immunity^29,30^. In melanoma, Tregs are heavily recruited, contributing to the formation of an immunosuppressive milieu^31^. A reduced CD8^+^ cytotoxic T cell/Treg ratio in melanoma patients is predictive for the diminished response to chemotherapy^32,33^ and poorer overall survival^34^. In this study, genistein inhibited melanoma growth and promoted tumor cell death. Notably, its therapeutic efficacy was remarkably potentiated when combined with a PD-1 inhibitor. Mechanistically, genistein reduced Treg abundance in the melanoma microenvironment and increased CD8^+^ cytotoxic T cell infiltration, leading to an elevated CD8^+^/Treg ratio. Besides, genistein treatment increased pro-inflammatory cytokines (IFN-γ and TNF-α) in tumor tissues and reduced Treg-derived IL-10 production. The combination of genistein and anti-PD-1 significantly amplified these immune effects, highlighting the critical role of checkpoint inhibitors in this synergistic therapy. These results indicated that genistein significantly reshaped the immune cell composition towards a pro-inflammatory phenotype.

Tregs exert immunosuppressive effects through multiple mechanisms, including secretion of inhibitory cytokines, attenuation of TCR-mediated signaling pathways, and suppression of pro-inflammatory molecules^35^. In vitro studies showed that genistein treatment dose-dependently inhibited IL-10 production by Tregs and downregulated mRNA expression of key regulatory proteins, such as Foxp3, CTLA-4, and LAG-3, which are critical for Treg differentiation and activation^36–39^. Based on these findings, we therefore investigated whether genistein directly impairs Treg function and differentiation.

The survival of Tregs is critically dependent on IL-2, which is primarily supplied by activated T cells^40^. Tregs express high levels of the IL-2 receptor α-chain (CD25), enabling them to competitively deplete IL-2 and induce functional exhaustion in neighboring cells^41^. IL-2 deprivation disrupts Th cell proliferation and reduces their cytokine production, including IFN-γ and TNF-α. Genistein pre-treatment eliminated Treg-mediated suppression of Th cell proliferation and cytokine secretion. Moreover, genistein significantly inhibited the in vitro differentiation of CD4^+^CD25^−^ naïve T cells into Tregs, as evidenced by a marked reduction in CD4^+^Foxp3^+^ Treg frequency and significant downregulation of Treg-related transcripts (Foxp3, CTLA-4, LAG-3 and IL-10).

PI3K/AKT signaling pathway is a critical axis for Treg development, homeostasis, and immunosuppressive function^42,43^. We observed that genistein treatment suppressed PI3K/AKT signaling during Treg differentiation, as indicated by reduced phosphorylation levels of PI3K and AKT. Pharmacological activation of AKT with SC-79 effectively restored AKT phosphorylation in genistein-treated induced T cells. SC-79 treatment also restored the differentiation potential of naïve T cells into Tregs, as evidenced by increased CD4^+^Foxp3^+^ Treg frequency and upregulation of Treg-related transcriptional signatures. These findings suggested that genistein inhibited Treg differentiation and function by suppressing the PI3K/AKT signaling pathway. Given that Tregs contribute to a unique metabolic profile to promote immunosuppression in the tumor microenvironment^44^, and PI3K/AKT signaling regulates cellular metabolic reprogramming^45^, future studies are warranted to clarify whether genistein disrupts Treg function through metabolic reprogramming.

A recent study reported contrasting results where genistein treatment showed no enhancement or even attenuated the efficacy of anti-PD-1 therapy in B16F1 melanoma, with no significant changes observed in tumor-infiltrating T cells or cytokine profiles^46^. The discrepancy between our findings and those reported might be attributed to several key differences in experimental design. While they used the B16F1 melanoma model, our study employed the more aggressive and metastatic B16F10 variant, which could explain the differential response to combination therapy. Additionally, the timing and dosing regimens of genistein administration differed between the studies, which may significantly impact immune modulation and therapeutic outcomes^46^. In addition, while our ELISA results showed reduced IL-10 levels in tumor lysates following combination treatment, we acknowledge that multiple cell populations within the tumor microenvironment can produce IL-10. Future studies using single-cell analysis would help distinguish whether the decreased IL-10 levels are primarily due to reduced tumor burden or reflect a fundamental shift toward a less immunosuppressive microenvironment.

Several limitations of our study warrant further investigation. First, while we observed significant therapeutic effects, the potential for tumor relapse after treatment termination needs to be evaluated through longer-term follow-up studies. Second, we focused on tumor-infiltrating Tregs, but understanding genistein’s effects on Tregs in other tissues would provide valuable insights into tissue-specific responses. Third, given that over 50% of melanoma patients harbor BRAF mutations, testing our therapeutic approach in BRAF-mutant models would provide more clinically relevant insights. Furthermore, previous studies have suggested genistein’s potential role in suppressing tumor invasiveness, which merits further investigation in our combination therapy context. While we focused on Treg modulation, future studies should explore genistein’s broader effects on melanoma-immune cell crosstalk, particularly its impact on tumor-associated neutrophils, tumor-associated macrophages, and myeloid-derived suppressor cells, to fully understand the comprehensive mechanisms underlying its therapeutic efficacy. Finally, future studies should investigate how genistein modulates PD-L1 expression and other immune checkpoint molecules in both tumor cells and immune cells, particularly comparing anti-PD-L1 monotherapy versus anti-PD-L1 + genistein combination therapy, to better understand potential resistance mechanisms and optimize therapeutic strategies.

## Conclusion

This study demonstrated that genistein exhibited antitumor effects and immunomodulatory potential in a mouse melanoma model. Besides, genistein enhanced the therapeutic efficacy of anti-PD-1 therapy by suppressing Treg-mediated immunosuppression and augmenting CD8^+^ T cell-mediated immunity. The inhibitory effects of genistein on Treg function and differentiation could be attributable to the suppression of the PI3K/AKT signaling pathway. Collectively, these findings provide preclinical evidence supporting genistein as a promising adjuvant for immunotherapy in melanoma. Since our study demonstrates the therapeutic potential of combining genistein with anti-PD-1 therapy, we envision that genistein could be developed as a standardized supplement to ensure consistent dosing and bioavailability for enhancing anti-PD-1 therapy. Based on our mechanistic findings regarding Treg modulation, patients with elevated Treg levels who show suboptimal response to anti-PD-1 monotherapy might be the most suitable candidates for this combination approach.

## Supplementary Information

Below is the link to the electronic supplementary material.

Supplementary Material 1

Supplementary Material 2

## Data Availability

The data generated in this study are included in this published article.
