# Supplementary material for: Genistein enhances anti-PD-1 efficacy in melanoma by suppressing regulatory T cell differentiation and activity
Source: Sci Rep. 2025 Oct 22;15:36973. doi: 10.1038/s41598-025-20941-7 (PMC12546575; doi:10.1038/s41598-025-20941-7)
Supplement: Supplementary file 1 — Supplementary Material 1 [file 41598_2025_20941_MOESM1_ESM.docx]

**Figure 3B**


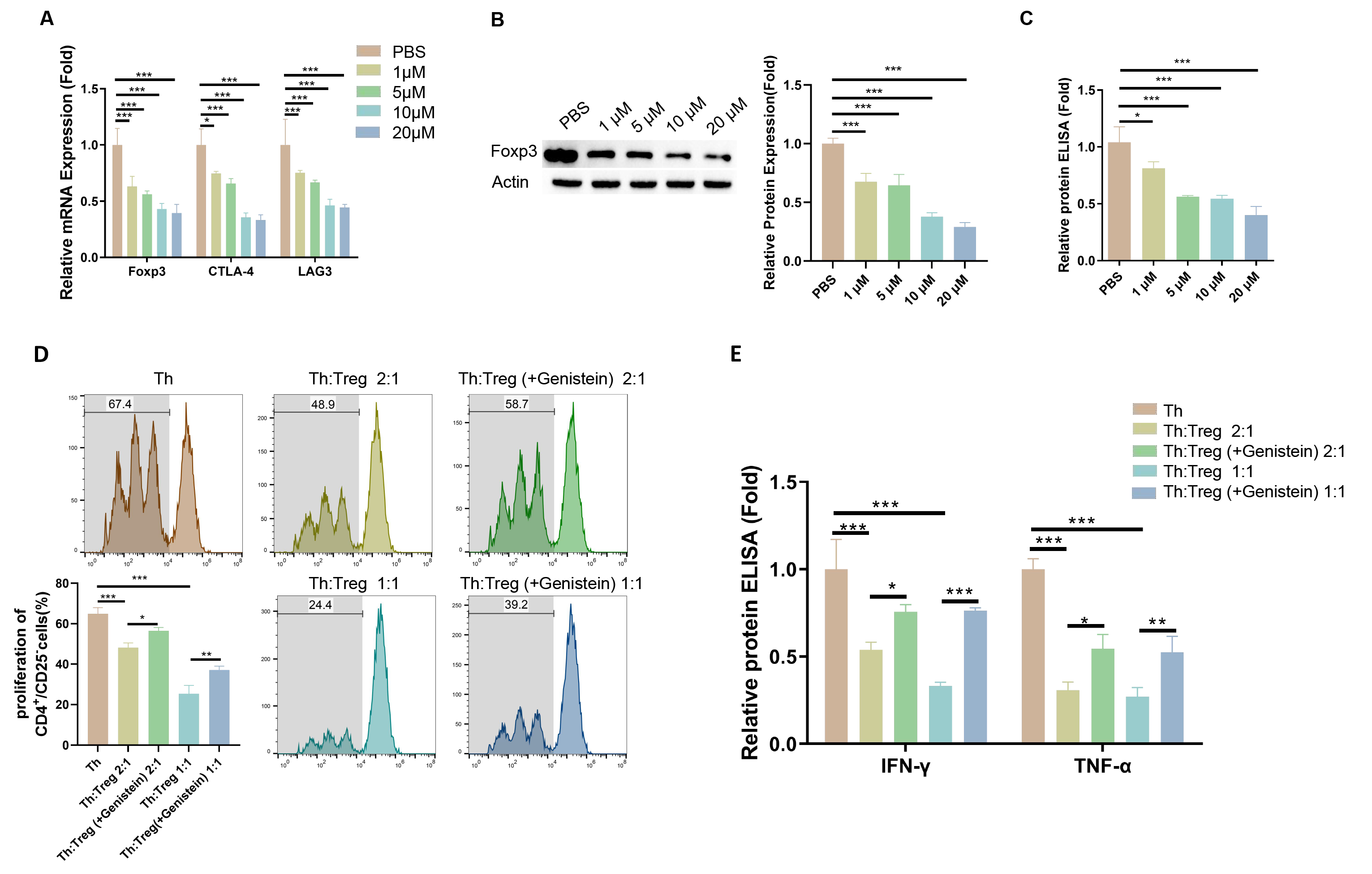


Actin

Exposed signal

Membrane with ladder


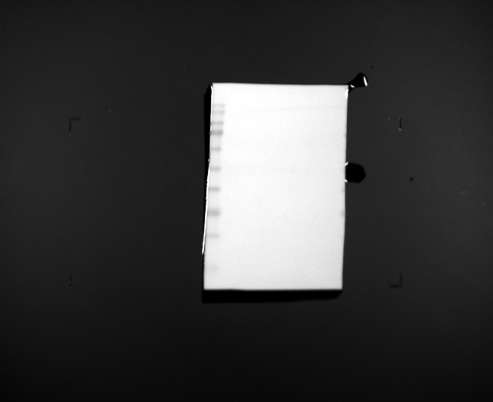

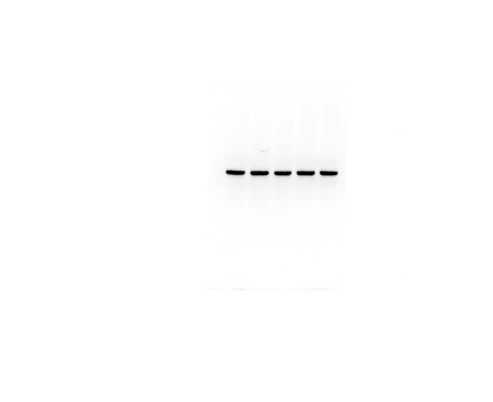

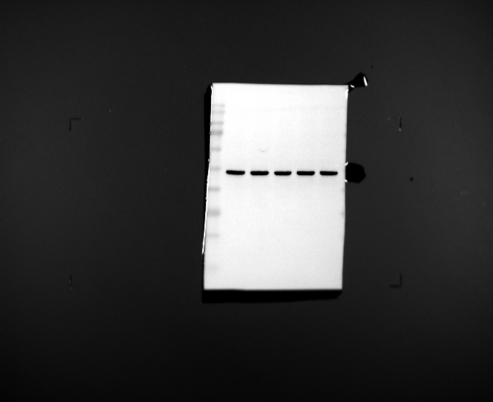


Molecular weight: 42 kDa

Merge

Foxp3

Exposed signal

Membrane with ladder


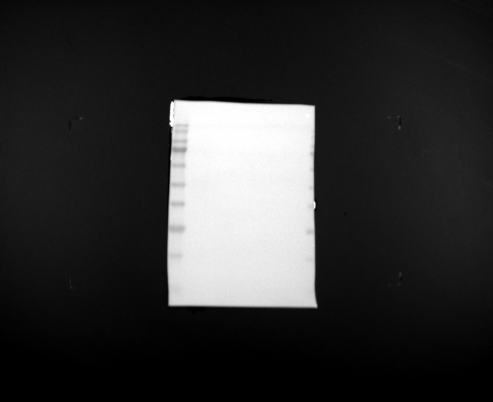

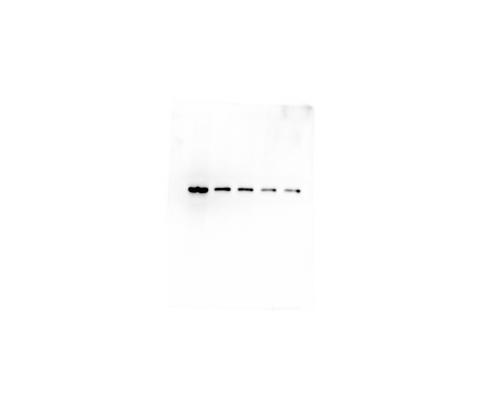

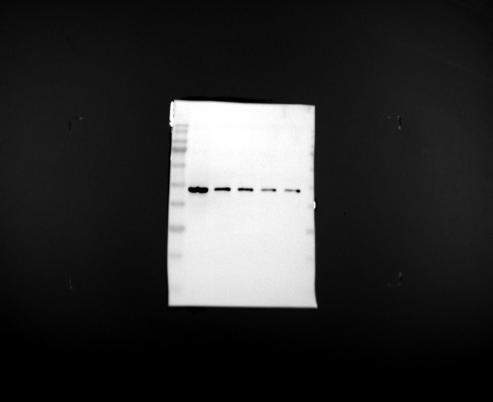


Molecular weight: 47 kDa

Merge

**Figure 4C**

**
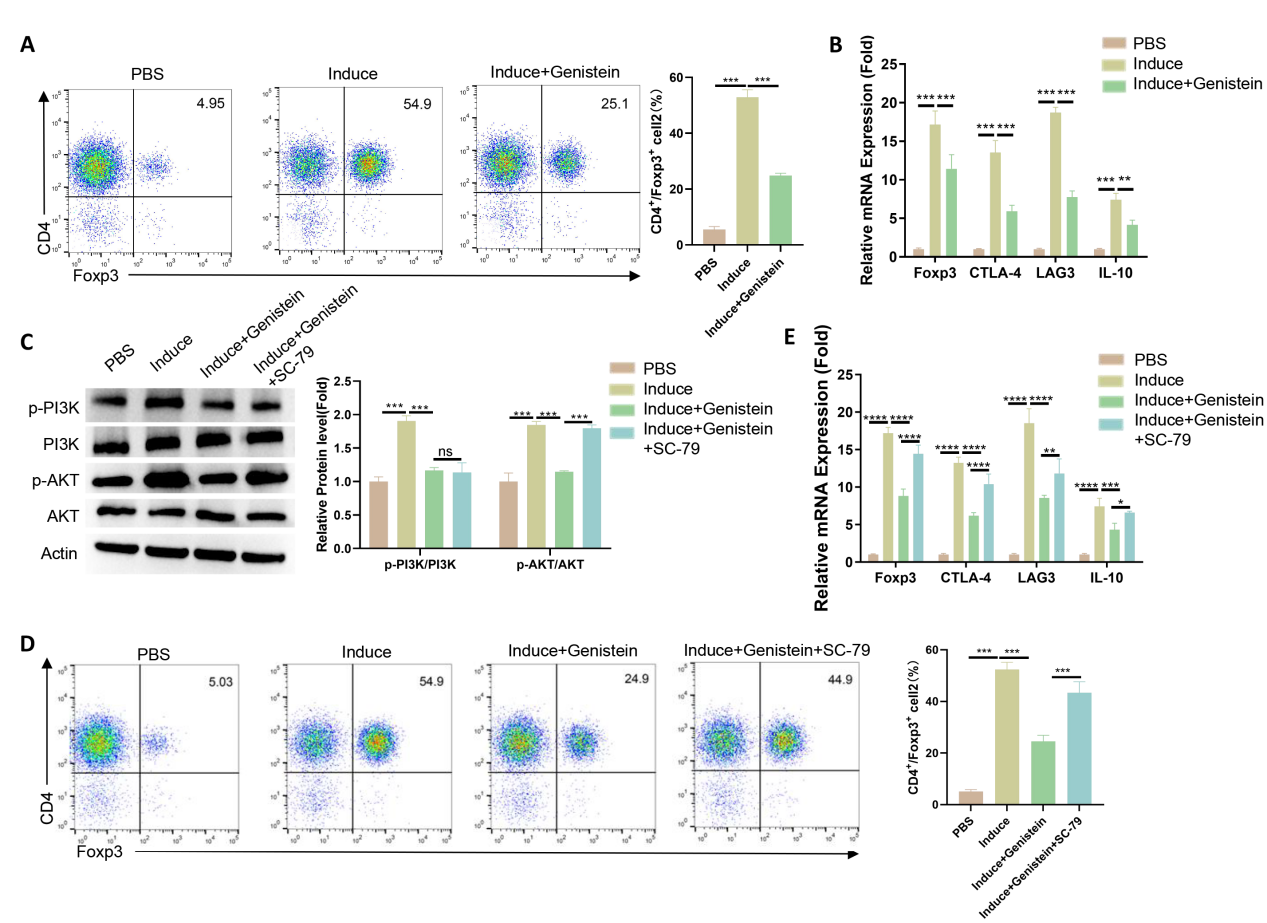
**

Actin

Exposed signal

Membrane with ladder


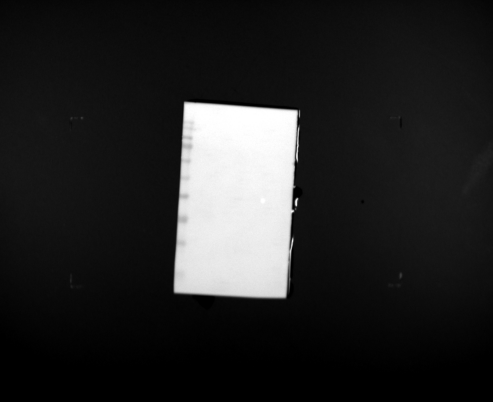

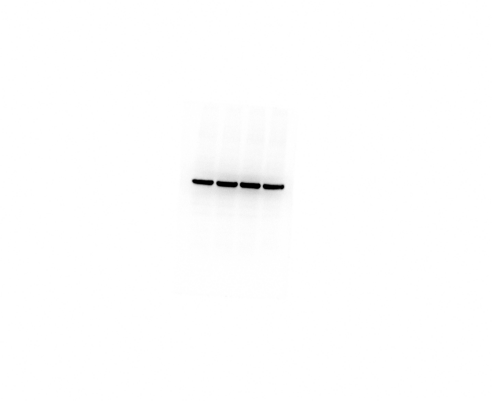

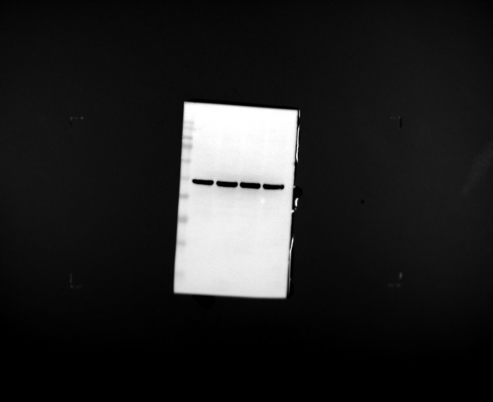


Molecular weight: 42 kDa

Merge

AKT

Exposed signal

Membrane with ladder


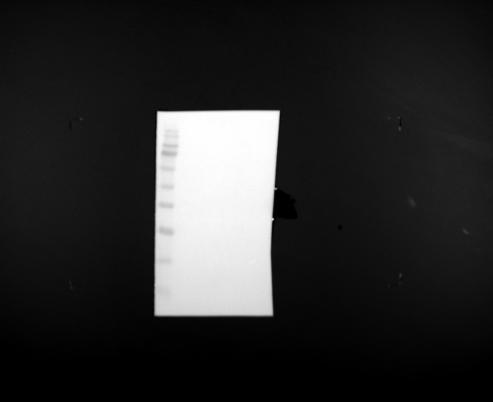

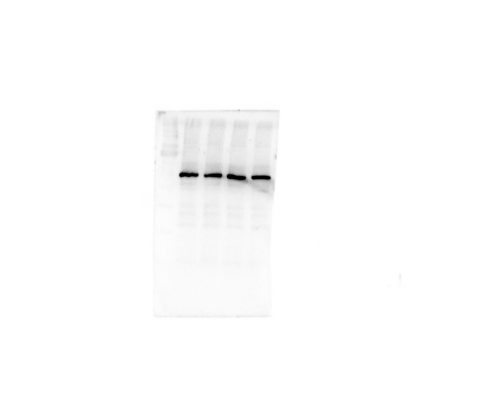

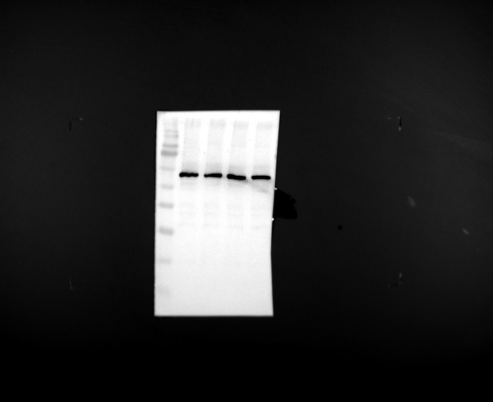


Molecular weight: 36 kDa

Merge

p-AKT

Exposed signal

Membrane with ladder


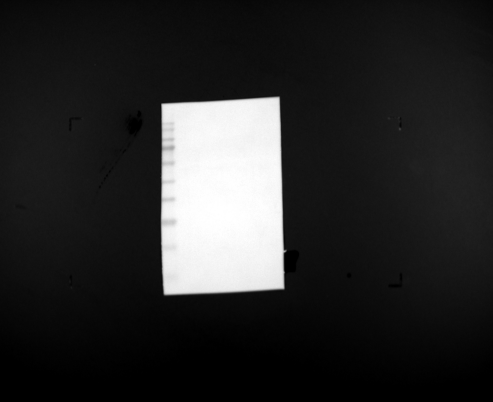

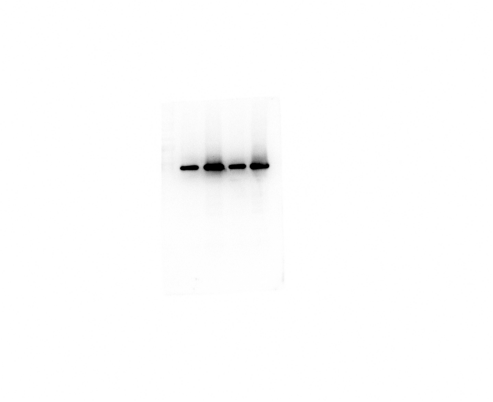

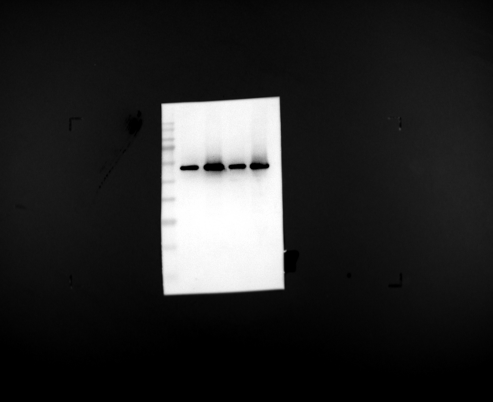


Molecular weight: 36 kDa

Merge

PI3K

Exposed signal

Membrane with ladder


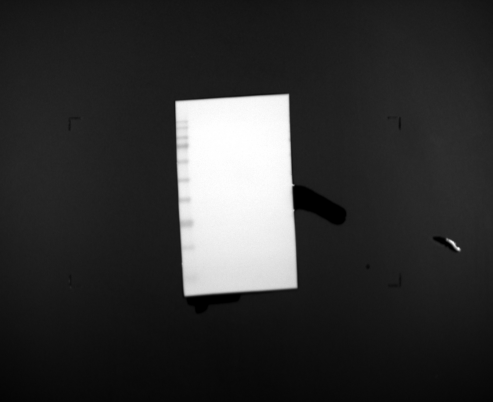

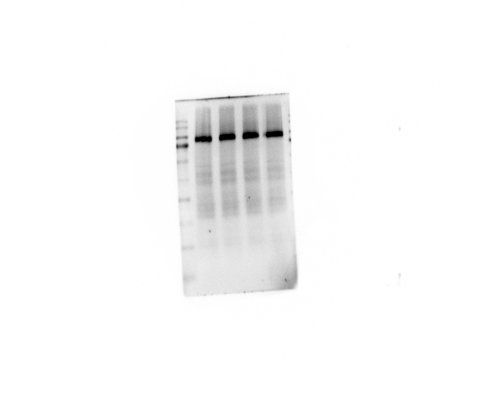

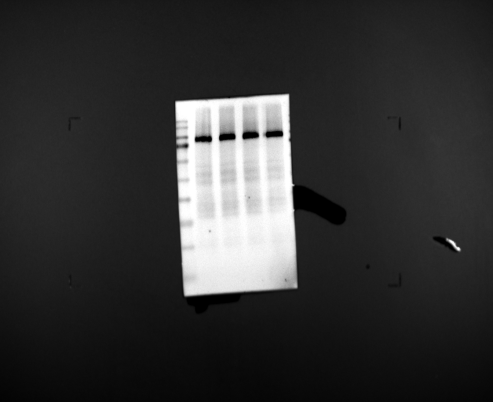


Molecular weight: 85 kDa

Merge

P-PI3K

Exposed signal

Membrane with ladder


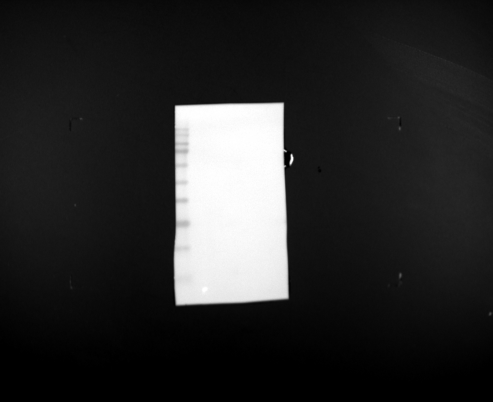

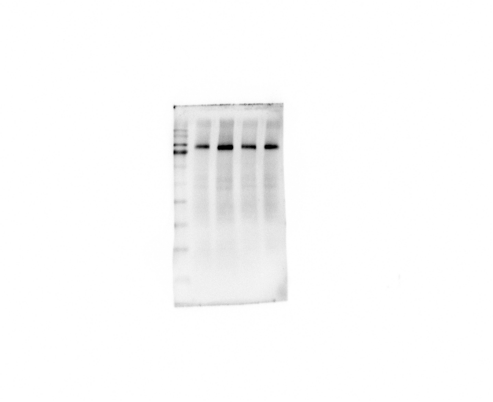

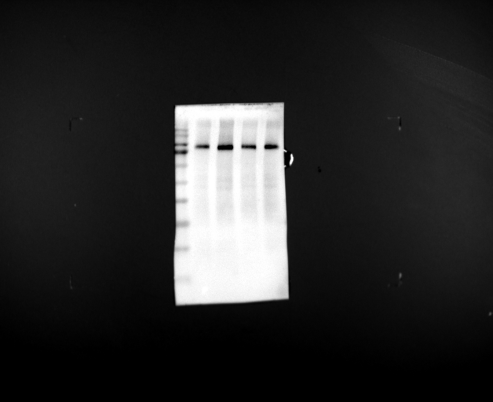


Molecular weight: 85 kDa

Merge
