# Supplementary figures and images for: Genistein enhances anti-PD-1 efficacy in melanoma by suppressing regulatory T cell differentiation and activity
Source: Sci Rep. 2025 Oct 22;15:36973. doi: 10.1038/s41598-025-20941-7 (PMC12546575; doi:10.1038/s41598-025-20941-7)

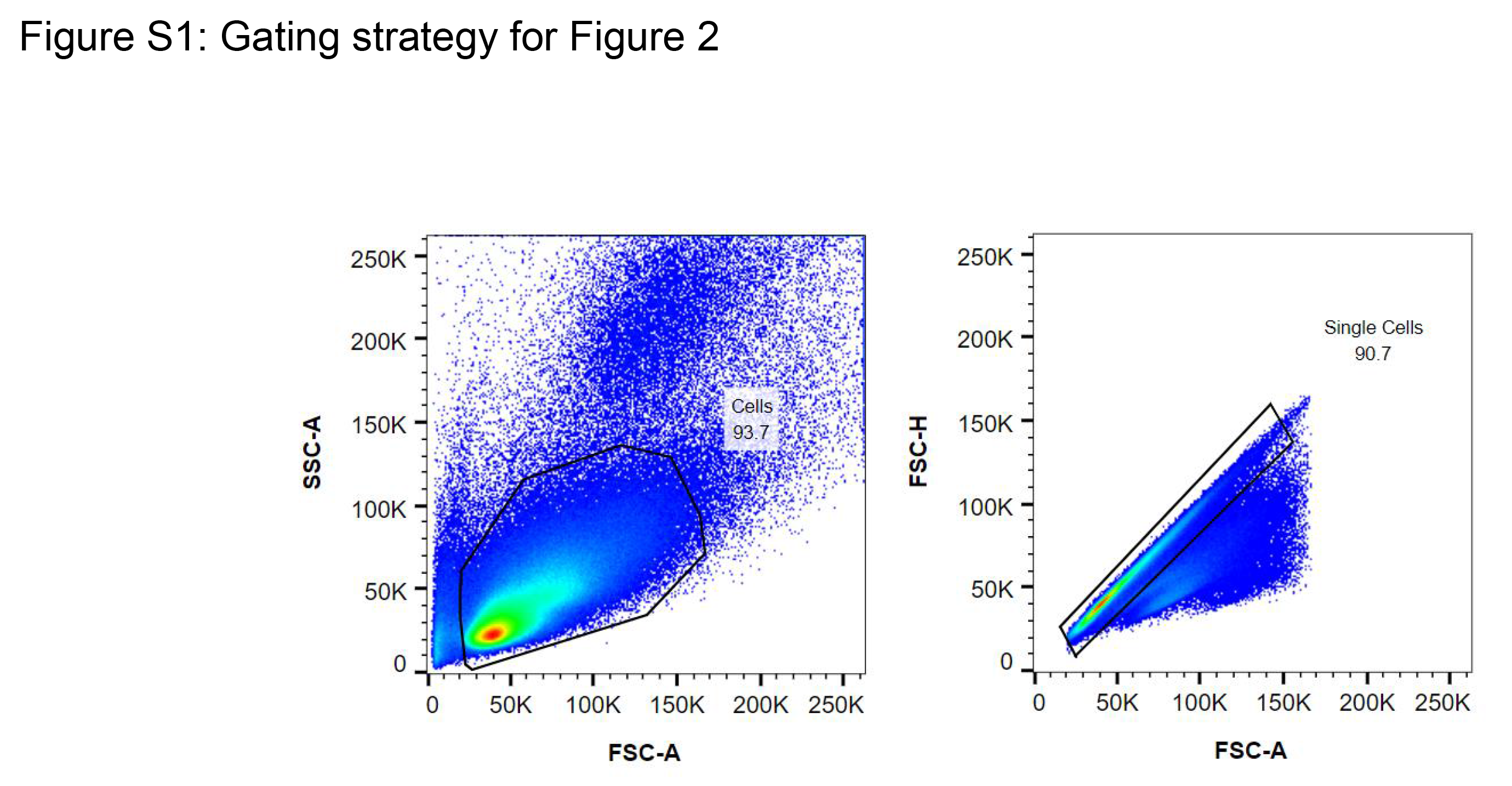

Supplement: Supplementary file 2 — Supplementary Material 2 [file 41598_2025_20941_MOESM2_ESM.tif]
